# Skin Grafting

**Published:** 1871-01

**Authors:** 


Skin Grafting.—Dr. J. J. Chisolm, in the *Richmond and Lou-*

*isville Medical Journal*, gives a description of this novelty in surgery, with records of cases in which he has practiced it. The process consists in the transplanting of epidermis into the raw surface of ulcers for the purpose of promoting their healing; it is, really, by engrafting a portion of epithelium in the sores, furnishing a new point of growth for epidermis.

It is a very common surgical experience to have a large ulcer heal kindly for a time, but, healing less rapidly each day, to finally cease entirely to lessen in size, and to baffle all attempts to induce healing. Epithelium can alone be produced by epithelium, and new integument to cover an ulcer grows only from the borders of that already existing; successive portions become less active and vital, and so it happens that after a while the reproduction ceases, and the cells on the borders of new surface tissue have just sufficient vitality to keep alive, but not enough to produce new cells. The reason why the old sores resist efforts to heal then is the absence of this procreative action.

By planting a few cells of healthy epithelium at various points in the unhealing surface of such ulcers, new centers of epithelial formation are established, which go on to the development of healthy skin, and form a complete covering for the raw surface.

This sound cicatrix of fully vitalized cells does not contract as much as is usual with the natural healing, and hence ugly deformities, such as come from extensive burns, may often be avoided.

The plan of M. Reoerdin is to snip out a small piece of healthy epithelium, with a pair of scissors, from the same trunk or thigh, of the size of half a rice grain. It should include some portion of the rete mucosum, as, if it consists entirely of the thin, horny scales of the epidermis, it will have no power to reproduce cells; in the rete mucosum the cells have the inherent power of re-producing thin skin when placed under favorable conditions.

In the operation of grafting, this little piece of tissue is thrust into a small puncture in the raw surface, and retained by adhesive straps.

These living cells very soon, absorbing pabulum from the blood and tissues, begin to proliferate, and the part perceptibly grows. It enlarges in every direction, and if the borders of the ulcer are not too far distant, or if a sufficient number of grafts have been made, they coalesce in time, with the inactive periphery, and the cicalrization is accomplished.

The rapidity of this process will depend somewhat on the age of the patient, as well as the number of points ingrafted; the younger the patient, the more rapid the growth.

Care should be used not to get too large a piece of tissue for the graft; and if it is thrust too deeply in the surface, it fails to take root, while, if too near the surface, it is washed away by the secretions of the part. Even when planted with care, all grafts do not grow.

It is usual that some days, often a week or two, must transpire before any good can be detected in the germs, and it is not until a month that they increase to the size of grains of corn.

